# Monoclonal Antibodies as Tools to Combat Fungal Infections

**DOI:** 10.3390/jof6010022

**Published:** 2020-02-04

**Authors:** Sebastian Ulrich, Frank Ebel

**Affiliations:** Institute for Infectious Diseases and Zoonoses, Faculty of Veterinary Medicine, Ludwig-Maximilians-University, D-80539 Munich, Germany; s.ulrich@lmu.de

**Keywords:** monoclonal antibodies, invasive fungal infections, therapy, prophylaxis, opsonization

## Abstract

Antibodies represent an important element in the adaptive immune response and a major tool to eliminate microbial pathogens. For many bacterial and viral infections, efficient vaccines exist, but not for fungal pathogens. For a long time, antibodies have been assumed to be of minor importance for a successful clearance of fungal infections; however this perception has been challenged by a large number of studies over the last three decades. In this review, we focus on the potential therapeutic and prophylactic use of monoclonal antibodies. Since systemic mycoses normally occur in severely immunocompromised patients, a passive immunization using monoclonal antibodies is a promising approach to directly attack the fungal pathogen and/or to activate and strengthen the residual antifungal immune response in these patients.

## 1. Introduction

Fungal pathogens represent a major threat for immunocompromised individuals [1]. Mortality rates associated with deep mycoses are generally high, reflecting shortcomings in diagnostics as well as limited and often insufficient treatment options. Apart from the development of novel antifungal agents, it is a promising approach to activate antimicrobial mechanisms employed by the immune system to eliminate microbial intruders. Antibodies represent a major tool to mark and combat microbes. Moreover, monoclonal antibodies (mAbs) are highly specific reagents that opened new avenues for the treatment of cancer and other diseases. This review provides an overview on studies in which mAbs have been used to combat experimental fungal infections caused by pathogenic yeasts, (*Candida*, *Cryptococcus*), dimorphic fungi (*Histoplasma*, *Paracoccidioides*, *Sporothrix*), or molds (*Aspergillus*, *Rhizopus*, *Scedosporium*).

## 2. Elimination of Microbial Pathogens by Antibody-Dependent Mechanisms

The antibody–antigen binding is a highly specific interaction that can directly modulate the biological activity of a target molecule, e.g., by neutralization of a toxin. Apart from secreted molecules, antibodies can also inhibit microbial surface proteins, such as adhesins or surface-bound enzymes. Surface-reactive antibodies can furthermore act as opsonins and thereby mark microbes out for destruction. Fcγ receptors reside in the cytoplasmic membrane of phagocytes and recognize bound IgGs. In concert with the parallel recognition of conserved microbial structures by dedicated pattern recognition receptors, this boosts phagocytosis, enhances phagosome–lysosome fusion, and results in a more efficient microbial killing [2]. Bound IgM or IgG can furthermore recruit complement proteins to activate this part of the innate immune response resulting in an enhanced C3 receptor-mediated phagocytosis. Moreover, antibodies can have a catalytic activity; as exemplarily shown by Bowen et al. [3]: Two mAbs directed against glucuronoxylomannan (GXM) the major component of the *Cryptococcus* capsule were shown to possess a proteolytic activity and one of them was additionally able to cleave the GXM oligosaccharide. A major advantage of this mode of action is that catalytic antibodies can directly harm their target organism and therefore act independently of other elements of the immune system.

## 3. The Cell Wall as Primary Target Structure for Antifungal Antibodies

Antigens must be accessible for antibodies; surface-bound molecules and secreted proteins are therefore particular suitable target molecules. In contrast to plant-pathogenic fungi, dedicated virulence factors are rare in fungi causing systemic mycoses in mammals. Structural components like the capsule of *Cryptococcus neoformans* or general attributes like the dimorphism of *Candida albicans* clearly contribute to pathogenicity, but hardly any proteins are known that specifically attack host cells or highjack parts of the host cellular machinery. Although pathogenic fungi release a plethora of proteases, lipases, and other enzymes, these proteins seem to be of limited importance for the virulence of most human-pathogenic fungi. Consequently, the vast majority of protective antibodies described so far recognize surface bound antigens.

After binding to surface antigens, antibodies can act as opsonins to boost the phagocytic activity of immune cells. The fungal cell wall represents the most important target structure for opsonizing antibodies; it contains proteins, but consists mainly of carbohydrate polymers. Due to the lack of appropriate T cell responses, most antibodies directed against carbohydrate antigens belong to the IgM class that cannot interact with Fcγ-receptors, but this drawback can be experimentally overcome by coupling glycoantigens to a carrier protein. A particular problem to the immune response is the ability of many fungi to switch between different morphotypes, since many antigens are expressed in a morphotype-specific pattern. Consequently, the immune system needs to employ multiple receptors and mechanisms to combat and eliminate these pathogens. Phagocytosis is a major antimicrobial mechanism, but phagocytes have a limited capacity with respect to the size of their pray. This poses another problem, but only for certain fungal morphotypes: Yeasts and other single cells are taken-up easily, while hyphae are protected simply by their size.

## 4. Protective Antibodies against *Cryptococcus neoformans*

*Cryptococcus neoformans* is a major yeast pathogen that is unique among medically important fungi in its possession of a polysaccharide capsule. While infections of healthy individuals usually remain asymptomatic, hosts with a severely impaired cellular immunity can develop life-threatening, disseminated infections and meningitis. In contrast to *C. albicans*, *Cryptococcus* does not form hyphae during infection making it a seemingly easier target for an antibody-based therapy.

The *Cryptococcus* polysaccharide capsule is a crucial virulence determinant with GXM being its major component. As for certain bacteria, the capsule prevents recognition by pathogen recognition receptors and thereby protects the fungus from phagocytes. However, as for capsulated bacteria, this can be overcome by antibody-mediated opsonization.

The first report of a mAb providing protection against experimental cryptococcosis dates back to 1987 [4]. In this pioneering study, Dromer and co-workers used a GXM-specific IgG_1._ Several years later, a similar protective activity was reported for a GXM-specific IgM [5]. Several studies directly compared GXM-specific mAbs belonging to different (sub)classes (Table 1A). The IgG_3_ subtype turned out to be less protective or even deleterious, whereas mice immunized by administration of IgG_1_, IgA or IgM antibodies showed an improved outcome [6,7]. In vitro experiments revealed no difference in the opsonizing activity of the different isotypes [8]. Yuan et al. provided evidence that the IgG_1_-mediated protection and the deleterious effect of IgG_3_ depend on CD4^+^- and CD8^+^-T cells, respectively [9]. A non-protective IgG_3_ could be converted into a protective IgG_1_ by isotype switching, indicating that the IgG_3_ subclass is a crucial determinant in this context [10]. Further studies implicated distinct Fcγ-receptor functions [6], the genetic background of the infected mice [11], and distinct catalytic activities [3] in the strikingly different biological activities of these GMX-specific IgG_1_ and IgG_3_ switch variants.

IgM antibodies to GMX can be either protective or non-protective, which depends on a variety of factors, e.g., the route of infection, the size of the inoculum, the amount of mAbs administered, and the ability of these antibodies to promote phagocytosis [12]. Further studies showed that protection requires binding to certain GXM epitopes [13,14,15]. Shapiro et al. showed that protection mediated by GXM-specific IgM antibodies is independent of complement component C3 indicating that complement fixation is not required [16].

Remarkably, high GXM-specific titers can also cause deleterious effects in mice and this was attributed to the formation of antibody-antigen complexes. Depending on the antibody titer and the inoculum, this antibody-mediated acute lethal toxicity (ALT) can be induced by different IgG subclasses [17]. *Cryptoccoccus* infections are often chronic and released capsular polysaccharides can accumulate to very high levels in tissue and serum. Antibody induced ALT depends on the antigen concentration in the blood and the isotype of the antibody. Data of two groups indicate that IgG_1_, IgG_2a_, and IgG_2b_ can be deleterious, while IgG_3_, IgM, and IgA lack this harmful activity [18,19]. ALT is triggered by the murine IgG_1_ 2H1, but is not induced by a mouse-human chimeric IgG_2_ derived from 2H1 suggesting that Fcγ-receptor binding is crucial for this toxic effect [20].

## 5. Protective Antibodies against *Candida albicans*

The first evidence that antibodies are important during candidiasis came from the finding that patients who survived systemic infections developed strong antibody responses to certain *C. albicans* proteins, whereas patients who succumbed to infection had no, minor or fading responses [21]. An immunodominant 45 kDa polypeptide was described and later on identified as a fragment of the heat shock protein Hsp90 [22]. Hsp90 is a highly conserved ATP-dependent molecular chaperone that stabilizes other molecules, governs morphogenesis, and is regarded as a key regulator of *Candida* virulence traits [23]. An IgG raised against *C. albicans* Hsp90 was the first mAb that was successfully tested in a murine model of systemic candidiasis [24]. Based on this murine immunoglobulin, a humanized, single chain antibody was developed, initially designated Efungumab, but later on renamed to Mycograb. This recombinant antibody is assumed to inhibit Hsp90 activity by binding to a central domain of Hsp90 that is responsible for the conformational change triggered by ATP binding [25]. Hsp90 is normally cytoplasmic, but to a certain extent, also a surface-bound protein [26]. It plays an important role in several stress responses including those triggered by antifungals. Accordingly, in vitro studies demonstrated a synergistic activity of Mycograb and antifungals, such as fluconazole, caspofungin, and amphotericin B. A clinical trial revealed that Mycograb plus lipid-associated amphotericin B produced significant clinical improvement for patients suffering from invasive candidiasis [27], but despite these promising results, marketing authorization was disapproved by the European Medicines Agency in 2017 based on concerns that the benefits of this treatment do not outweigh its risks.

A different approach was taken by Torrosantucci et al. [28], who coupled the β-glucan laminarin to the diphtheria toxoid and obtained polyclonal antibodies that defended mice against infections caused by *C. albicans* or *A. fumigatus*. A monoclonal β-glucan specific IgG2b antibody obtained by this approach and designated 2G8 turned out to be protective against *C. albicans*, *A. fumigatus*, and *C. neoformans* infections [29,30]. Remarkably, an IgM harboring the same complementarity-determining region as 2G8 was not protective [30]. Further experiments revealed that the IgG2b was highly specific for β-1,3-glucan and showed a much stronger reactivity with β-glucan molecules that are released by *C. albicans* than the corresponding IgM. These distinct specificities may explain the strikingly different protective potential of both antibodies. Interestingly, the IgG_2b_ was also reported to inhibit growth of *C. albicans* and *A. fumigatus* in vitro, but the precise mode of action was not determined. More recently, a mouse–human chimera and a scFv-Fc derived from 2G8 were shown to promote killing of *C. albicans* by isolated neutrophils and to protect mice in a vulvovaginal model of infection [31]. In 2019, Matveev et al. reported that an IgG_1_ specific for β-1,3-glucan delayed germination of *A. fumigatus* conidia and improved survival of mice infected with *C. albicans* via the intravenous route [32].

In a series of papers, Han, Cutler, and colleagues characterized two IgM mAbs directed against different *C. albicans* cell wall components. In immunofluorescence, both antibodies recognized the yeast, but not the hyphal form. B6.1, which is specific for β-1,2-linked mannotriose, stained *Candida* yeast cells more homogenously than B6 [33]. Both B6.1 and B6 protected mice against vaginal *C. albicans* infections [34], whereas only B6.1 provided protection in a model of disseminated candidiasis [35]. An IgG_3_ mAb also recognizing β-1,2-linked mannotriose was later on shown to be protective in both the disseminated and the vaginal infection model and this was attributed to the strong complement binding mediated by IgG_3_ immunoglobulins [36].

Using a mannan-specific humanized IgG_1_ antibody, Zhang et al. observed enhanced phagocytosis of *C. albicans* by murine macrophages, increased deposition of complement component C3, and protection of mice from an otherwise lethal dose of *C. albicans* yeast cells [37]. Moreover, generation of recombinant switch variants of this antibody revealed that an IgG_2_ variant was less protective than the corresponding IgG_1_, IgG_3_, or IgG_4_ immunoglobulins [38].

A fully humanized IgG_1_ specific for β-1,6-linked poly-N-acetyl-D-glucosamine, a capsular antigen of several bacterial pathogens, cross-reacts with *C. albicans* and protected mice in a *Candida* keratitis model [39]. Kavishwar and Shukla described another protective antibody that belongs to the IgA isotype and binds to glycosyl moieties of *C. albicans* proteins [40].

Several other studies analyzed the impact of antibodies directed against different *Candida* surface proteins. In these experiments, IgG_1_, IgG_3_, and IgM mAbs provided protection in different models of infection [41,42,43,44]. One of these mAbs, designated C7, is directed against the Als3 mannoprotein, which has multiple functions, e.g., as an adhesin and invasin [45]. In vitro studies with this IgM demonstrated a direct growth inhibitory activity [46] that was later on attributed to an antibody-mediated inhibition of fungal iron acquisition [47].

Polyclonal rabbit antibodies directed against the surface-bound Hyr1 protein of *C. albicans* were also shown to be protective in a murine model of infection [48] and more recently, Rudkin et al. characterized several recombinant IgG_1_ mAbs specific for Hyr1 and other *C. albicans* surface molecules that were derived from B cells isolated from human patients [49]. These mAbs enhanced phagocytosis of *C. albicans* yeasts and short hyphae by murine macrophages and protected mice from a systemic *C. albicans* infection. Probably, these human IgG_1_ antibodies interact with murine Fcγ receptors and thereby boost the antifungal activities of macrophages and neutrophils. While the Hyr1-specific antibodies recognized exclusively *C. albicans*, the mAbs directed against other surface proteins were also reactive with other members of the genus *Candida* and may therefore possess a broader therapeutic potential [49].

The minor role of secreted proteins in fungal virulence in mammals has been mentioned above. However, de Bernardis et al. showed that antibodies against the secreted aspartic protease Sap2 are protective in a rat model of vaginitis, thereby demonstrating that protection is not restricted to mAbs directed to surface antigens [41]. Since Sap2 plays an important role in vaginal infections caused by *C. albicans* [50], it is conceivable that the protective activity of this mAb is due to inhibition of the proteolytic activity of Sap2.

Killer toxins (KTs) have been described for *Saccharomyces* spp., *Pichia* spp., and other non-pathogenic yeasts. These short, secreted proteins bind to the surface of sensitive fungi and kill them through different effector mechanisms [51]. According to the concept of anti-idiotypic antibodies, an antibody raised against an immunoglobulin specific for the active site of a particular enzyme can possess the enzymatic activity of this enzyme. Using this approach, anti-idiotypic recombinant antibodies were generated that mimic the antifungal activity of a KT derived from *Wyckerhamomyces anomalus* (formerly *Pichia anomala*). This recombinant antibody killed *C. albicans* in vitro and provided protection in a rat model of *C. albicans* vaginitis [52]. Interesting features of KT-like antibodies are their direct antifungal activity and their target structures that are often conserved in many fungal pathogens.

In summary, most antibodies that are protective against *C. albicans* or *C. neoformans* infections recognize different glycostructures or surface proteins (Table 1A,B). They are either IgMs or belong to one of the four IgG subclasses. Many protective antibodies were shown to enhance the phagocytic uptake and stimulate the phagolysosomal maturation process (Table 2). In all *C. albicans* protection experiments that mimic a systemic infection (Table 1B), the yeast form was injected intravenously and in most cases, the antibodies were given prior to infection. Hence, the yeast cells are immediately opsonized and rapidly eliminated by phagocytes in the blood stream. However, this setting does not reflect the normal sequence of events associated with a systemic *C. albicans* infection. The yeast form is often present in the blood stream, but it is normally not the dominant morphotype during infection; hyphae are more abundant and spread in the infected tissue. Whether the protective antibodies described so far are also able to attack *C. albicans* hyphae and thereby to provide protection in naturally acquired cases of invasive candidiasis remains to be determined. Moreover, for those antibodies that were shown to inhibit hyphal growth in vitro, it is, in most cases, unclear how this growth repression is achieved. More research is clearly required to address these issues.

## 6. Protective Antibodies against Dimorphic Fungi

Dimorphic fungi are a family of six fungal pathogens of humans mainly found in the Americas that show a unique temperature-induced morphological transition: They grow in their filamentous form in the environment but switch to the yeast morphotype during infection. Several studies explored the therapeutic use of antibodies in infections caused by *Histoplasma capsulatum*, *Paracoccidioides brasiliensis*, and *Sporothrix schenckii.* Opsonization of *H. capsulatum* by an IgM directed to a surface-bound, histone 2b-like protein promoted the anti-fungal activity of macrophages and resulted in a faster maturation and stronger acidification of their phagosomes [82]. Moreover, administration of this antibody to *Histoplasma*-infected mice reduced the fungal burden, decreased pulmonary inflammation, and prolonged survival [59]. The heat shock protein Hsp60 is a major and protective *H. capsulatum* T cell antigen. Hsp60-specific antibodies of the IgG_1_ and IgG_2a_, but not of the IgG_2b_ subclass reduced the intracellular survival in macrophages, increased phagolysosomal fusion, and prolonged the lives of infected mice [60]. In contrast, an IgG_1_ directed against a 70 kDa surface protein of *H. caspulatum* surprisingly increased the intracellular fungal growth and reduced macrophage nitric oxide release in vitro but had no effect on fungal burden or survival in a murine model of infection [61]. Another promising surface protein is the so-called M antigen of *H. capsulatum*. Opsonization with three M antigen-specific mAbs (one IgM and two IgG2a) resulted in enhanced phagocytosis and provided full protection in experimental murine histoplasmosis [62].

In a more recent study, Liedke et al. generated a chitin-specific, recombinant antibody-chimera consisting of the chitin-binding domain of the lectin wheat germ agglutinin (WGA) and the Fc portion of a murine IgG_2a_. Only 10 µg of WGA-Fc were sufficient to elicit full protection in mice that received a normally lethal dose of *H. capsulatum* [81]. In vitro, WGA-Fc triggered increased phagocytosis and complement deposition and thereby promoted an efficient elimination of the pathogen. Remarkably, WGA-Fc also bound to *C. albicans* and *C. neoformans* and sparked an enhanced killing of these pathogens by murine macrophages. Due to this cross-reactivity, WGA-Fc is a promising candidate for the development of a pan-fungal therapeutic [81].

In *P. brasiliensis*, several surface glycoproteins are well-known diagnostic antigens. Passive transfer of mAbs directed against gp70, gp43, and a 75 kDa secreted phosphatase proved to be protective in murine models of infection [63,64,65] and the same applies to mAbs directed against the heat shock protein 60 of *P. lutzii* [67]. A different approach was taken by Ferreira et al., who constructed a single-chain variable fragment (scFv) antibody derived from the antiidiotypic antibody 7.B12 [66]. This recombinant construct resembles the internal image of gp43 and thereby served as a substitute for this antigen. When expressed in dendritic cells that were administered to mice, it triggered an enhanced T cell response, elevated levels of anti-gp43 antibodies, and a dramatic reduction in the number of viable fungi. In a subsequent study, the same group demonstrated that the protective effect could be further enhanced if the scFv molecules were incorporated into poly(lactide-co-glycolic) acid nanoparticles [85].

Another approach that targeted glycoproteins was undertaken with *Sporothrix schenckii*, a fungus causing chronic subcutaneous mycosis in humans and animals. An IgG_1_ raised against a 70 kDa glycoprotein and putative adhesin protected mice from this pathogen when administered either before, during, or even three days after infection [68,69]. A humanized version of this IgG_1_, given 3d post infection, also reduced the fungal burden in the spleens, but not in livers of infected mice [70].

## 7. Protective Antibodies against Molds

Molds are a heterogeneous group of soil-dwelling fungi that share a common lifestyle. Their asexual spores are efficiently spread in the environment, whereas hyphae, their vegetative morphotype, grow in the soil and other habitats. *Aspergillus fumigatus* is currently the most frequent mold causing severe mycoses, but infections caused by Mucorales and other filamentous fungi are recognized with increasing frequencies. As mentioned above, the filamentous growth of these pathogens during infection represents a particular challenge for the immune system.

In a first study, Frosco et al. analyzed five mAbs specific for a so-called elastase of *A. fumigatus* that all turned out to be non-protective [71]. Cenci et al. reported a first successful passive immunization experiment with *A. fumigatus* using an antiidiotypic mAb representing the internal image of yeast killer toxin [72]. This mAb also inhibited the hyphal growth in in vitro experiments. A similar growth inhibition and protection was later on reported for 2G8, a mAb specific for β-1,3-glucan [30]. As for *Candida*, a corresponding IgM sharing an identical binding site with 2G8 was non-protective. Another abundant and homogenously distributed glycostructure present on *Aspergillus* hyphae is galactomannan, but a galactomannan-specific IgM failed to provide protection in mice infected intravenously [75]. These data fit well to the more recent finding that an efficient killing of *A. fumigatus* hyphae by neutrophils requires antibody-mediated opsonization and activation of Fcγ-receptors through binding of suitable IgG antibodies [86].

However, other studies provided evidence that IgM can be protective against *A. fumigatus* infections. An IgM initially raised against sialyl-lacto-N-tetraose of B group streptococci was shown to recognize a glycoantigen present on *A. fumigatus* conidia and hyphae. After passive transfer, this mAb protected mice infected with *A. fumigatus* via the intravenous or intratracheal route [76]. The elimination of *A. fumigatus* conidia and germ tubes by human neutrophils was previously shown to depend on antibody-mediated complement activation [87]; as IgM binds complement factors, protection is most likely established by activation of the classical complement pathway.

Another IgM directed against enolase, an enzyme of the glycolytic pathway, strongly inhibited *Aspergillus* hyphal growth and prolonged survival of intravenously infected mice [78]. Enolase is one of the so-called moonlighting proteins; it normally resides in the cytoplasm, but some molecules are also found on the cell surface. In vitro experiments showed that the enolase-specific IgM had a striking growth inhibitory activity on *Aspergillus* hyphae [78], but the underlying mechanisms have not been defined yet. Appel et al. coupled an IgM recognizing a cell wall antigen of *A. fumigatus* to alliinase, an enzyme that converts the harmless garlic compound allicin to alliin, a substance with a broad antifungal activity. If administered together with allicin, this conjugate was able to protect mice from *A. fumigatus* infections [74].

Two further studies analyzed mAbs directed against protein antigens. An IgG_1_ recognizing an *A. fumigatus* cell wall glycoprotein inhibited the growth and even killed *A. fumigatus* hyphae in vitro. Moreover, this antibody substantially increased the survival times in a murine model of infection [73]. However, due to the lack of follow-up studies, the identity of the antigen and the antifungal mode of action employed by this antibody remained undefined. Chauvin and co-workers generated a humanized IgG_1_ antibody directed against Crf1, an *Aspergillus* cell wall enzyme with transglycosylase activity [77]. The Crf1 protein is a prominent T cell antigen providing striking cross-protection against *A. fumigatus* and *C. albicans* [88]. The IgG_1_ antibody detected Crf1 on the hyphal surface, both in vitro and in vivo, inhibited the enzymatic activity of Crf1 and caused a slight growth retardation of *A. fumigatus* hyphae in vitro. However, when tested in a rat model of infection, this antibody failed to provide protection [77].

Mucorales are a group of non-septated, filamentous molds representing another severe threat for immunocompromised patients. So far, mucormycoses are less frequent than *Aspergillus* infections, but the numbers have clearly increased in recent years and these rapidly progressing infections are particular difficult to treat [89]. The CotH3 protein of *Rhizopus delemar* resides on the fungal surface and its interaction with the human glucose-regulated protein (GRP) 78 represents a key event in the hyphal invasion of endothelial cells. Loss of CotH3 results in attenuated virulence [84] and polyclonal CotH3-specific antibodies were shown to block the interaction between CotH3 and GRP78 and thereby reduce invasion of an endothelial layer. These antibodies were furthermore able to inhibit the growth of *R. delemar* in in vitro experiments. The monoclonal anti-CotH3 antibody designated C2 had similar activities and was successfully used in protection experiments with intratracheally infected mice [79]. Protection was mediated by binding of the Fc part of the C2 IgG_1_ immunoglobulin to the corresponding Fcγ-receptor. This interaction triggered enhanced opsonophagocytosis and thereby limited the infection. Application of the antibody in combination with either posaconazole or amphotericin B amplified the protective effect and saved all infected animals [79].

A remarkable example for a mAb causing an exacerbated infection was reported for *Scedosporium proliferans*. This IgG_1_ directed to surface-bound peptidorhamnomannan enhanced fungal germination, impaired phagocytosis by macrophages, and reduced the survival time of infected mice [80]. The authors speculated that binding of this mAb modifies certain activities of the fungus and thereby enhances its virulence.

## 8. Conclusions

A large number of studies have provided evidence that the passive transfer of antibodies can protect animals from fungal infections. However, these studies summarized in Table 1A–D are difficult to compare for several reasons: (i) The different pathogenic fungi have a variety of distinct features that are decisive for the respective infections and this can influence the biological impact of therapeutic antibodies, e.g., shedding of capsular polysaccharides by *C. neoformans* is the reason for ALT. (ii) The biological properties of immunoglobulins differ significantly, e.g., their ability to interact with Fcγ-receptors or to fix complement depend on their (sub)class and the animal species they are derived from. (iii) The routes of experimental infections differ and do not always reflect the natural infection processes. (iv) In a patient, therapeutic antibodies will be given when the infection process has already progressed to a level causing clinical symptoms, but in most studies, antibodies were given prior to infection, which reflects a prophylactic rather than a therapeutic use. (v) The amount of antibody is a critical factor and varies in murine studies at 10–1000 µg per animal. Up to now, only few studies compared different amounts of a given antibody to determine an optimal dosage.

**Figure 1:**
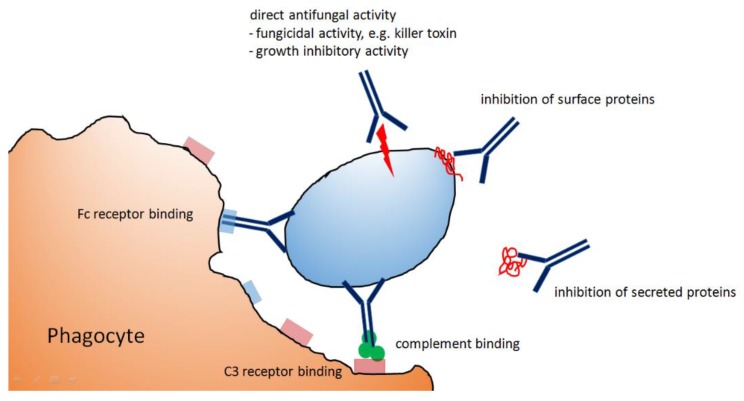
Different modes of actions employed by antibodies to inhibit or kill fungal pathogens.

The protective impact of a certain antibody clearly depends on the mechanisms exerted to eliminate the fungal pathogen. The major mechanisms employed by antibodies in order to harm fungal pathogens are schematically depicted in Figure 1. Opsonization can result in an efficient elimination of small and predominantly unicellular fungi, and it can boost other antimicrobial effector mechanisms, e.g., by attraction and activation of neutrophils. Another mechanism reported by several studies is the antibody-mediated inhibition of fungal growth, but our knowledge about suitable antigen/antibody combinations and the underlying molecular processes is still in its infancy. Antibodies with a direct and deleterious impact on the fungus represent a particularly promising option, since they act independently of other immune molecules and cells, which is an obvious advantage in a severely immunocompromised host. Antibodies may inhibit the biological function of surface proteins and thereby reduce the ability of the target cell to adapt to certain stress situations (e.g., anti Hsp90 mAbs). Alternatively, antibodies may interfere with transport channels, uptake systems, or proteins that are required for the maintenance and reorganization of the cell wall. Anti-idiotypic antibodies employ a direct antifungal mode of action or may alternatively act as a substitute for the original antigen; this can boost an immune response directed towards this antigen and thereby provide protection. Secreted proteins are in principle attractive targets, but up to now, only one mAb specific for *C. albicans* Sap2 was shown to be protective in models of *Candida* vaginitis. Antibodies against conserved cell wall glycostructures can bind to a range of fungal pathogens. The use of chimeric molecules, such as the lectin domain-containing WGA-Fc construct, can extend the repertoire of suitable molecules, but a potential drawback of this strategy is that the lectin domain may trigger a strong immune response that could prevent a prolonged application.

The serious threat posed by invasive fungal infections is a persisting problem and therefore new therapeutic options are clearly required. Monoclonal antibodies are now widely used in modern medicine, but we are just beginning to explore their potential in the context of fungal infections. The data available so far that are summarized here strongly suggest that mAbs are promising prophylactic tools, but further studies are clearly required to determine whether the same applies to a therapeutic use in the setting of an already established fungal infection.

## Figures and Tables

**Table:** (**A**)

Antigen	Infection Model	Application of mAbs ^#^	Protective	Non-Protective	Reference
GXM	m., i.v.	i.p./24 h/10–100 µg	IgG_1_		[4]
capsular polysaccharide	m., i.v.	i.p./−24 h/1 mg i.p./48 h +96 h/500 µg		IgG_1_ *, IgG_2a_, IgG_2b_ * IgG_1_ *, IgG_2a_, IgG_2b_ *	[53]
GXM	m., i.p.	i.p./0 h/1 mg	IgA, IgG_1_ > IgM > IgG_3_		[7]
GXM	m., i.v.	i.p./−4 h/1 mg	chIgG_1_		[54]
GXM	m., i.p.	i.p./−15 min/1 mg	two IgM	one IgM	[13]
GXM	m., i.v.	i.p./−24 h/1 mg	IgG_1_, IgG_2b_, IgG_2a_ > IgG_3_		[6]
GXM	m., i.v.	preincubation of yeasts with mAb	hIgM		[5]
melanin	m., i.v.	i.v./−30 min/1 mg	IgM		[55]
GXM	m., i.v. m., i.p. m., i.t.	i.p./−5, −30 min/0.1, 0.5 or 1 mg i.p./−5, −30 min/0.1, 0.5 or 1 mg i.p./−5, −30 min/0.1, 0.5 or 1 mg	IgM 12A1, dose-dependent	IgMs 12A1 and 13F1 IgM 13F1 IgMs 12A1 and 13F1	[12]
GXM	m., preincubation of yeasts	i.p./−24 h/1 mg	IgG_1_ *, IgG_2a_ *, IgG_2b_ *	IgG_3_ *	[16]
GXM	m., i.p.	i.p./−1 h/0.5, 5, 50, 100, 1000 µg	one hIgM (at 100 µg)	two hIgM	[14]
GXM	m., i.v.	i.v./10 d/500 µg	chIgG_2_		[20]
GXM	m., i.v.	i.p./−18 h/0.1–1 mg	recomb. h-IgG_2_ *, h-IgG_4_ *	recomb. h-IgG_1_ *, h-IgG_3_ *	[56]
glucosylceramide	m., i.t.	i.p./−24 h/100, 250, 500 µg	IgG_2b_ at 500 µg	IgG_2b_ at 100 and 250 µg	[57]
β-glucan	m., i.v.	i.p./−2 h, +1 d/200 µg	IgG_2_		[29]
GXM M2 motif	m., i.v.	i.p./−30 min/500 µg		IgA, IgM	[15]

**Table:** (**B**)

Antigen	Infection Model	Application of mAbs ^#^	Protective	Non-Protective	Reference
Hsp90	m., i.v.	i.v./−1 h/740 µg	IgG		[24]
β-1,2-linked mannotriose	m., i.v.	i.p./−4 h, 20 h/125 µg	IgM		[35] [35]
polysaccharide mannoprotein SAP2	r., i.vg. r., i.vg. r., i.vg.	i.v./30 min/100 µg/mL i.v./30 min/100 µg/mL i.v./30 min/100 µg/mL	IgM IgG_1_	IgG_1_	[41]
β-1,2-linked mannotriose	m., i.vg.	i.p./−4 h, 24 h /35 µg, 10 µg i.vg./−4 h, 24 h/35 µg, 10 µg	IgM IgM		[34]
antiidiotypic KT antibody	r., i.v.	i.v./0 h/10 µg	single chain antibody		[52]
β-1,2-linked mannotriose	m., i.v. m., i.vg.	i.p./−4 h/125 µg i.vg./−4 h/10 µg	IgM, IgG_3_		[36]
Hsp90	m., i.v.	i.v./2 h/2 mg/kg	recomb. h-IgG		[25]
PRA1 (mannoprotein 58)	m., i.v.	i.p./−2 h/1.8 mg	IgG_1_		[42]
β-1,3-glucan	m., i.v.	i.p./−2 h/250 µg	IgG_2_		[28]
ALS3 mannoprotein	m., i.v.	i.p./−4 h, 1 d, 2 d/200, 100, 100 µg	IgM		[43]
mannan	m., i.v.	i.p./−4 h/63 µg–4 mg	h-IgG_1_		[37]
cell wall carbohydrate	m., i.v.	i.v./−2 h/100 µg	IgA		[40]
β-1,3-glucan	m., i.v.	i.p./−2 h/100 µg	IgG_2_ *	IgM *	[30]
Fba peptide	m., i.v.	i.p./−4 h/8 µg	IgM		[58]
β-1,3-glucan	m., i.v. r., i.v.	i.p./−2 h/100 µg i.v./1 h + 24 h + 48 h/50 µg	scFv-Fc		[31]
PNAG	m., keratitis model	i.p./24 h/200 µg	hIgG_1_		[39]
Fba peptide Met6 peptide	m., i.v. m., i.v.	i.p./−4 h, every day/100 µg i.p./−4 h. every day/250 µg	IgM IgG_3_		[44]
mannan	m.,	i.p./−4 h/1 mg	hIgG_1_, hIgG_3_, hIgG_4_	hIgG_2_	[38]
Unknown surface antigen HYR1 protein	m., i.v. m., i.v.	i.p./−4 h/1 mg i.p./−4 h/1 mg	h-IgG_1_ h-IgG_1_		[49]
β-1,3-glucan	m., i.v.	i.p./−2 h/150 µg	IgG_1_, IgG_3_		[32]

**Table:** (**C**)

Antigen	Infection Model	Application of mAbs ^#^	Protective	Non-Protective	Reference
***Histoplasma capsulatum***					
histone 2b-like protein	m., i.n.	i.p./−2 h/100 µg	IgM		[59]
Hsp60	m., i.n.	i.p./−2 h/500 µg	IgG_1_ **, IgG_2a_	IgG_2b_ **	[60]
70 kDa surface protein	m., i.n.	i.p./−2 h/100–500 µg		IgG_1_	[61]
chitin	m., i.n.	i.p./−2 h/10 µg	WGA-Fc (IgG_2a_)		[62]
***Paracoccidioides brasiliensis***					
glycoprotein of 70 kDa (gp70)	m., i.t.	i.v./−3 d, 3 d, 6 d, 9 d, 42 d/100 µg each	combination of two IgG_1_		[63]
75 kDa secreted phosphatase	m., i.t.	i.v./−3 d/100 µg	IgG, IgM		[64]
glycoprotein of 43 kDa (gp43)	m., i.t.	i.p./30 d/1 mg	IgG_2b_		[65]
gp43	m., i.t.	i.m./14 d, 21 d/DCs expressing the scFv s	scFv		[66]
	m., i.t.				
***Paracoccidioides lutzii***					
Heat shock protein 60	m., i.t.	not sp./−24 h/1 mg	IgG_2a_, IgG_2b_		[67]
***Sporothrix schenckii***					
70 kDa glycoprotein	m., i.p.	i.p./−24 h, 3 d, 6 d, 42 d/100 µg	IgG_1_		[68]
70 kDa glycoprotein	m., i.p.	i.p./3 d, 10 d/100 µg	IgG_1_		[69]
70 kDa glycoprotein	m., i.p.	not sp./3 d/100 µg	hIgG_1_		[70]

**Table:** (**D**)

Antigen	Infection Model	Application of mAbs ^#^	Protective	Non-Protective	Reference
***Aspergillus fumigatus***					
elastase	m., i.n.	i.p./4 h/50 µg		isotype not sp.	[71]
antiidiotypic KT antibody	m., i.n.	i.n./each day/2 × 1 µg	rat IgM		[72]
cell wall glycoprotein	m., i.v.	i.v./−2 h/50 µg	IgG_1_		[73]
unknown cell wall antigen	m., i.n.	i.t./1 h/50 nmol	IgM + alliinase		[74]
galactomannan	m., i.v.	i.p./−15 min/200 µg		IgM	[75]
sialylated oligosaccharides	m., i.v. m., i.t.	i.v/0 min/200 µg i.t./0 min/50 µg	IgM		[76]
Crf1 protein	r., i.t.	i.t./4 mg/kg/0 h + 32 h	h-IgG_1_		[77]
enolase	m., i.v.	i.v./2 h/50 µg	IgM		[78]
***Rhizopus delemar***					
CotH3 protein	m., i.t.	i.p./48 h/30 µg	IgG_1_		[79]
***Scedosporium apiospermum***					
peptidorhamnomannan	m., i. t.	i.p./−2 h/250 µg		IgG_1_	[80]

In (**A**) ^#^: route of application/time point of application relative to the time point of infection/amount of mAbs. *: identical complementarity-determining regions, **: mapped to the same epitope. m = mouse, r = rat, h = humanized, i.m = intramuscular, i.t. = intratracheally, i.v. = intravenous, i.vg. = intravaginal, not sp. = not specified. chIg = chimeric mouse-human immunoglobulin, KT = killer toxin, PNAG = β-1,6-poly-*N*-acetyl-d-glucosamine, scFv = single-chain variable fragment, MET6 = 5 methyltetrahydropteroyltriglutamate homocysteine methyltransferase, Fba = fructose-bisphosphate aldolase.

**Table 2:** Activities triggered by selected anti-fungal antibodies in vitro.

Antibody	Antigen	Subclass	Fungus	Antifungal Activity	Mode of Action	Reference
Mycograb	Hsp90	rec. mAb	*Candida albicans*	stress resistance ↓	inhibition of Hsp90	[25]
C7	ALS3 mannoprotein	IgM	*Candida albicans* *Candia lusitaniae* *Cryptococcus neoformans* *Aspergillus fumigatus* *Scedosporium proliferans*	growth inhibition, adhesion to HEp2 cells ↓	reduced iron acquisition	[46] [47]
2G8	β-glucan	IgG_2b_	*Candida albicans*	growth inhibition	unknown	[28,30]
G5	cell wall carbohydrate	IgA	*Candida albicans*	growth inhibition	unknown	[40]
5H5	β-1,3-glucan	IgG_3_	*Candida albicans* *Aspergillus fumigatus*	growth inhibition, phagocytosis ↑	unknown, osponization	[32]
M1g1	mannan	h-IgG_1_	*Candida albicans*	phagocytosis ↑, killing ↑	complement binding ↑	[37]
2G8 scFv-Fc	β-glucan	scFv-Fc	*Candida albicans*	neutrophil mediated killing ↑	osponization	[31]
6D2, 11B11	melanin	IgM	*Cryptococcus neoformans*	growth inhibition	unknown	[55]
12A1	glucuronoxylomannan	IgM	*Cryptococcus neoformans*	phagocytosis ↑	opzonisation	[12]
recomb. 3E5	GXM	IgG_1_, IgG_3_	*Cryptococcus neoformans*	phagocytosis ↑	opzonisation	[56]
recomb. 3E5	GXM	IgG_1_, IgG_3_	*Cryptococcus neoformans*	phagocytosis ↑	opzonisation	[8]
2G8	β-glucan	IgG_2b_	*Cryptococcus neoformans*	growth inhibition, phagocytosis ↑	unknown,	[29]
WGA-Fc	chitin	(IgG_2a_)	*Cryptococcus neoformans*	growth inhibition, phagocytosis ↑	unknown, opsonization	[81]
4E12	Hsp60	IgG_2a_	*Histoplasma capsulatum*	phagocytosis ↑	opsonization	[60]
9C7	histone 2b-like protein	IgM	*Histoplasma capsulatum*	phagocytosis ↑, phagosomal maturation ↑	opsonization	[59] [82]
MS112-IIB1	Crf1, glycosylhydrolase	hum. IgG_1_	*Aspergillus fumigatus*	growth inhibition	inhibition of enzymatic activity	[77]
R-5	enolase	IgM	*Aspergillus fumigatus*	growth inhibition	unknown	[78]
7	catalase B	IgM	*Aspergillus fumigatus*	growth inhibition	unknown	[83]
2G8	β-1,3-glucan	IgG_2b_	*Aspergillus fumigatus*	hyphal growth ↓, adherence to epithelial cell ↓	unknown	[28,30]
3G11	β-1,3-glucan	IgG_1_	*Aspergillus fumigatus*	inhibition of germination, phagocytosis ↑	unknown, opsonization	[32]
C1, C2, C3	CotH3 protein		*Rhizopus delemar*	phagocytosis ↑, cytokine response ↑	opsonization	[84]
3E	Gp43	IgG_2b_	*Paracoccidioides brasiliensis*	phagocytosis ↑, NO ↑, IFNγ ↑	opsonization	[65]
1G6, 5E7C	75 kDa phosphatase	IgG, IgM	*Paracoccidioides brasiliensis*	phagocytosis ↑, growth inhibition	opsonization	[64]
7B6, 4E12	Hsp60	IgG_2a_, IgG_2b_	*Paracoccidioides lutzii*	phagocytosis ↑	opsonization	[67]
P6E7	Gp70		*Sporothrix* spp.	phagocytosis ↑	opsonization	[70]

↓ = reduced, ↑ = enhanced.
